# Transcriptome analysis of hypothalamus and pituitary tissues reveals genetic mechanisms associated with high egg production rates in Changshun green-shell laying hens

**DOI:** 10.1186/s12864-023-09895-0

**Published:** 2023-12-20

**Authors:** Wenbin Xu, Ren Mu, Tuya Gegen, Tiantian Ran, Qi Wu, Di Wen, Fen Wang, Zhi Chen

**Affiliations:** 1 College of Biological Science and Agriculture, Qiannan Normal University for Nationalities, Jianjiang Road 5, Duyun, 558000 China; 2 Qiannan Key Laboratory of Applied Biotechnology for Livestock and Poultry, Duyun, 558000 China; 3 College of Animal Sciences, Zhejiang University, Hangzhou, 310058 China; 4 Library, Qiannan Normal University for Nationalities, Duyun, 558000 China

**Keywords:** Transcriptome analysis, Hypothalamus, Pituitary, Egg production, Changshun green-shell laying hens

## Abstract

**Background:**

Changshun green-shell laying hens are unique to the Guizhou Province, China, and have high egg quality but relatively low yield. Egg production traits are regulated by the hypothalamus-pituitary-ovary axis. However, the underlying mechanism remains unclear. Thus, we conducted RNA sequencing of hypothalamic and pituitary tissues from low- and high-yielding Changshun green-shell laying hens to identify critical pathways and candidate genes involved in controlling the egg production rate.

**Results:**

More than 39 million clean reads per sample were obtained, and more than 82% were mapped to the *Gallus gallus* genome. Further analysis identified 1,817 and 1,171 differentially expressed genes (DEGs) in the hypothalamus and pituitary, respectively. Nineteen DEGs were upregulated in both the hypothalamus and pituitary of high-yielding chickens. The functions of these DEGs were mainly associated with ion transport or signal transduction. Gene set enrichment analysis revealed that the pathways enriched in the hypothalamus were mainly associated with gonadotropin-releasing hormone (GnRH) secretion, neurotransmitter release, and circadian rhythms. The pathways enriched in the pituitary were mainly associated with GnRH secretion, energy metabolism, and signal transduction. Five and four DEGs in the hypothalamus and pituitary, respectively, were selected randomly for qRT-PCR analysis. The expression trends determined via qRT-PCR were consistent with the RNA-seq results.

**Conclusions:**

The current study identified 19 DEGs upregulated in both the hypothalamus and pituitary gland, which could provide an important reference for further studies on the molecular mechanisms underlying egg production in Changshun green-shell laying hens. In addition, enrichment analysis showed that GnRH secretion and signal transduction, especially neurotransmitter release, play crucial roles in the regulation of egg production.

**Supplementary Information:**

The online version contains supplementary material available at 10.1186/s12864-023-09895-0.

## Introduction

Chicken eggs are an important human food resource because of their high nutritional value and low cost. According to data from the FAO, global egg consumption has witnessed impressive growth over the past 30 years, with an increase from 36.4 million tons in 1991 to 88.1 million tons in 2021, and this trend is predicted to continue in the future [1]. Improving the genetic potential of chickens is one of the most important strategies for increasing egg production. Thus, the molecular mechanisms underlying egg production must be further explored.

The hypothalamus-pituitary-ovary (HPO) axis is a synchronized communication network among the hypothalamus, pituitary, and ovary, and it is commonly considered to play a key role in chicken ovulation cycle and egg production via multiple pathways. For instance, the hypothalamus can secrete gonadotropin-releasing hormone (GnRH) into the hypophyseal portal circulation, causing the pituitary to synthesize and release gonadotropins, including luteinizing hormone (LH) and follicle-stimulating hormone (FSH). These gonadotropins act on the ovary to stimulate oogenesis and sex steroid hormone secretion [2]. In addition, other hormones and neuropeptides secreted by the HPO axis, such as gonadotropin-inhibitory hormone (GnIH), growth hormone (GH), and prolactin, have also been found to regulate the reproduction cycle [3]. In recent years, employing high-throughput techniques, more candidate genes and pathways that may be involved in the regulation of egg production have been identified [4]. Wang and Ma [5] sequenced the hypothalamus and pituitary expression profiles of high- and low-yielding laying Chinese Dagu chicken and revealed that egg production was strongly correlated with genes involved in amino acid metabolism, glycosaminoglycan biosynthesis, and the estrogen negative feedback system in the HPO axis. Mishra et al. [2] compared the transcriptomes of the hypothalamus, pituitary, and ovary of Luhua chickens selected for high and low egg production and identified 10, 414, and 356 differentially expressed genes (DEGs) in the hypothalamus, pituitary, and ovary, respectively. These DEGs were mainly involved in the regulation of the mTOR signaling pathway, Jak-STAT signaling pathway, tryptophan metabolism, and PI3K-Akt signaling pathways in the HPO axis. Using RNA-seq and WGS, Cai et al. [6] found eight candidate genes that might be responsible for the egg production performance of chickens. Taken together, the regulation of the HPO axis on chicken egg-laying performance is an integrated and complex process, and an improved understanding of this process is required.

Changshun green-shell chicken is a native breed found in Guizhou Province, China, and it produces eggs with extremely high economic value but at a relatively low yield [1]. We have previously analyzed the ovarian transcriptome of Changshun green-shell chicken and identified candidate genes involved in controlling egg production in ovarian tissue [1]. In the present study, we compared the transcriptomes of the hypothalamus and pituitary, the other two tissues of the HPO axis, between low- and high-yielding Changshun green-shell laying hens. The results of the present study are expected to provide further insights into the molecular mechanisms underlying egg production in Changshun green-shell chicken.

## Materials and methods

### Ethics statement

This study was carried out in accordance with the recommendations in the Guide for the Care and Use of Laboratory Animals of the Ministry of Science and Technology of the People's Republic of China (2017#676), the Regulations for the Administration of Affairs Concerning Experimental Animals, Qiannan Normal University for Nationalities (2020#3, Guizhou, China), and ARRIVE 2.0 guidelines.

### Animal and sample preparation

Details of the animal and sample preparation process are described in our previous study [1]. Briefly, 80 Changshun green-shell layers with similar body weights of 1.36 ± 0.14 kg were used in this study. The study lasted 60 days (from age of 240 days to 300 days), and the egg number and egg weight were recorded every day (16:00). At the end of the study, four high-yield (HY, laying rate of 93.67 ± 7.09%) and low-yield (LY, laying rate of 68.00 ± 5.56%) individuals were selected from the batch of laying hens (average laying rate of 76.03 ± 2.49%). After overnight fasting, the chickens were euthanized with an overdose of sodium pentobarbital, and then hypothalamic and pituitary samples were obtained. The samples were immediately frozen in liquid nitrogen and stored at -80 °C until analysis.

### Animal management

During the study, all layers were housed individually in battery cages (36 cm width × 48 cm length × 38 cm height) with the same feeding and management conditions. The room temperature was maintained at 22 ± 2 °C. The light regime was 16L:8D. The layers were allowed ad libitum access to food and water.

### RNA extraction, cDNA library construction, and mRNA sequencing

Methods used for RNA extraction, cDNA library construction, and mRNA sequencing are described in our previous study [1]. Total RNA was extracted from the hypothalamic and pituitary samples using the TRIzol reagent (Takara Bio, Dalian, China). The concentration and quality of total RNA were determined using a Nanodrop ND-2000 spectrophotometer (Thermo Scientific, Wilmington, DE, USA) and electrophoresis. Sample integrity was evaluated using a microfluidic assay on a Bioanalyzer (Agilent Technologies, Inc., Santa Clara, CA, USA). mRNA was enriched using magnetic beads with oligo (dT) and randomly fragmented using a fragmentation buffer. First-strand cDNA synthesis was performed with a random hexamer primer using the mRNA fragments as a template. Second-strand cDNA synthesis was then performed using buffer, deoxynucleotide triphosphates (dNTPs), ribonuclease H (RNase H), and DNA polymerase I. cDNA was purified using a QiaQuick PCR extraction kit (Qiagen, Germany) and eluted with elution buffer for end repair and poly (A) addition. Sequencing adapters were ligated to the 5′ and 3′ ends of the fragments. The fragments were purified by agarose gel electrophoresis and enriched by PCR amplification to obtain cDNA libraries, which were loaded onto an Illumina sequencing platform (NovaSeq 6000) for sequencing.

### Data analysis

Details of data analysis are described in our previous study [1]. Quality control checks for raw reads were performed using FastQC (v0.11.5). Raw reads were trimmed using fastx_trimmer (fastx_toolkit-0.0.13.2) to obtain clean reads, which were subsequently mapped against the chicken reference genome *Gallus gallus* (GRCg7b) available in Ensembl v98 using HiSAT2 (v2.2.1) with default parameters. Raw gene counts were obtained using the htseq-count package (v0.12.3) in Python (v3.5) and then normalized using the DESeq2 package (v1.28.1) in R (v4.0.2) to obtain gene expression levels.

### Identification of differentially expressed genes (DEGs)

DEGs were identified using the DESeq2 package (v1.28.1) in R (v4.0.2). Genes with an adjusted *p*-value ≤ 0.05, and |log_2_fold change|≥ 1 were considered differentially expressed.

### Gene set enrichment analysis

Kyoto Encyclopedia of Genes and Genomes (KEGG, http://www.genome.jp/kegg/) and Gene Ontology (GO, http://geneontology.org) gene set enrichment analyses (GSEAs) of the DEGs were performed using the clusterProfiler package (v3.16.1) in R (v4.0.2), with an adjusted *p* < 0.05 as the screening standard.

### Gene expression analysis by qRT-PCR

The mRNA expression values of eight randomly selected candidate genes were analyzed to verify the RNA sequencing results. β-Actin was used as an internal control for the normalization of expression levels. The primers used in the qRT-PCR were designed using Primer 5 (Table 1). The total RNA was reverse transcribed into complementary DNA (cDNA) using the Prime Script RT reagent Kit (Takara Bio, Dalian, China). Gene expression was analyzed using the ABI7900 system (Applied Biosystems, USA) and the AceQ qPCR SYBR Green Master Mix (Vazyme Biotech Co., Ltd, China). The PCR protocol was initiated at 95 °C for 10 min, followed by 40 cycles of the amplification program, with denaturation at 95 °C for 15 s, and annealing/extension at 60 °C for 60 s. Melt curves were generated at the end of the last amplification cycle to confirm the specificity of the amplification reaction. We carried out each assay in triplicate and included a negative control. Relative quantification of gene expression was performed using the 2^−ΔΔCt^ method.

**Table 1:** Primers used for qRT-PCR

Gene Symbol	Gene Name	Primer Sequence (5’-3’)	Accession Number
MBP	myelin basic protein	F:GCTTCACAAAAACGCTCCTC	NM_205280.1
		R:CCTGGCTGCGTGTATATCCT	
SPCS2	signal peptidase complex subunit 2	F:GGCTGCTCGATAAGTGGAAG	XM_417247.5
		R:GAGACACGAGATGGTGCAGA	
PGM2L1	phosphoglucomutase 2 like 1	F:TATTCACTGGCAACGAGCTG	XM_001233128.5
		R:CATTTGAAGCCAGGGAGTGT	
SLC2A13	solute carrier family 2 member 13	F:TTCTGCCCCACTCCATACTC	XM_001232939.5
		R:GTGCTTCTAGCCCACAGAGG	
LHX8	LIM homeobox 8	F:CTCCAGTCACAGCAGCTCAG	NM_001040466.3
		R:CAAAGGCTGGAGTCCAAGAG	
BG8	MHC B-G antigen	F:GGGATGGTCTCTCTGATGGA	NM_001030670.1
		R:TGACCCAACCAGAAGTGTGA	
ESR1	estrogen receptor 1	F:CTGGGCAAAGAGAGTTCCAG	NM_205183.2
		R:GATTTCCACCATGCCCTCTA	
AGTPBP1	ATP/GTP binding protein 1	F:AAAACAGGCATTGGTTACCG	NM_001305107.1
		R:CAAATGTCTGTCCCAACACG	
β-actin	beta-actin	F: GAGAAATTGTGCGTGACATGA	NM_205518.1
		R: CCTGAACCTCTCATTGCCA	

### Statistical analysis

Statistical analyses were performed using the R software (v4.0.2, R Development Core Team 2019). Data were analyzed using t-tests after testing for homogeneity of variance with the Levene’s test. All data are presented as the mean ± SD, and a *p-value* < 0.05 indicated significance.

## Results

### RNA sequencing quality assessment

The quality metrics of the transcriptomes are listed in Table 2. Sixteen cDNA libraries were constructed from the hypothalamus and pituitary, and more than 39 million raw and clean reads were obtained from each library. The GC content of all samples ranged from 49.20% to 51.52%, the Q20 base percentage was above 97.30%, and the Q30 base percentage was above 92.54%. All quality metrics did not differ significantly (*p* > 0.05) between groups.

**Table 2:** Quality metrics of transcripts

Sample	Raw reads	Clean reads	Clean bases	Q20 (%)	Q30 (%)	GC (%)	N (ppm)
LY-H1	42,072,616	41,922,716	6,237,389,514	97.60	93.34	49.20	4.65
LY-H2	39,739,476	39,595,258	5,884,694,753	97.81	93.82	50.81	4.69
LY-H3	40,472,092	40,290,928	5,989,704,692	97.30	92.74	49.87	5.68
LY-H4	46,677,586	46,512,088	6,918,963,075	97.65	93.44	49.53	6.97
HY-H1	50,886,106	50,699,212	7,538,118,641	97.53	93.29	51.30	4.64
HY-H2	44,681,150	44,564,184	6,631,653,990	97.73	93.55	50.65	7.24
HY-H3	43,872,014	43,736,682	6,503,963,076	97.74	93.62	50.00	5.79
HY-H4	41,897,238	41,767,616	6,215,250,424	97.67	93.46	49.68	4.67
LY-P1	41,783,508	41,626,586	6,193,266,068	97.63	93.51	50.11	4.54
LY-P2	42,456,388	42,255,300	6,278,301,281	97.18	92.54	49.81	6.91
LY-P3	40,732,838	40,585,448	6,037,953,383	97.83	93.99	50.49	4.72
LY-P4	41,737,220	41,598,832	6,187,567,990	97.65	93.51	49.88	4.76
HY-P1	45,547,430	45,354,578	6,741,697,201	97.45	93.17	51.52	4.70
HY-P2	43,867,158	43,711,718	6,501,101,496	97.60	93.41	50.08	4.73
HY-P3	45,297,464	45,166,294	6,720,396,746	97.70	93.52	49.52	4.64
HY-P4	40,795,166	40,658,890	6,043,789,907	97.61	93.39	50.18	5.69

### Transcriptome alignment

The results of trimming and read mapping are shown in Table 3. The total mapped ratio between the reads and the reference genome of all the samples ranged from 86.77% to 90.10%, and the uniquely mapped ratio ranged from 82.51% to 86.38%. The total mapped ratio and the uniquely mapped ratio did not differ significantly (*p* > 0.05) between groups.

**Table 3:** Summary of trimming and read mapping results

Sample	Total reads	Total mapped	Uniquely mapped	Multiple mapped
LY-H1	41,922,716	36,926,924 (88.08%)	35,551,390 (84.80%)	1,375,534 (3.28%)
LY-H2	39,595,258	35,229,068 (88.97%)	32,886,325 (83.06%)	2,342,743 (5.92%)
LY-H3	40,290,928	35,606,256 (88.37%)	33,531,832 (83.22%)	2,074,424 (5.15%)
LY-H4	46,512,088	41,565,115 (89.36%)	39,865,473 (85.71%)	1,699,642 (3.65%)
HY-H1	50,699,212	44,787,261 (88.33%)	42,808,891 (84.44%)	1,978,370 (3.90%)
HY-H2	44,564,184	39,969,715 (89.69%)	38,478,029 (86.34%)	1,491,686 (3.35%)
HY-H3	43,736,682	39,266,122 (88.78%)	37,694,238 (86.18%)	1,571,884 (3.59%)
HY-H4	41,767,616	37,519,023 (89.83%)	36,052,116 (86.32%)	1,466,907 (3.51%)
LY-P1	41,626,586	36,805,422 (88.41%)	35,282,184 (84.76%)	1,523,238 (3.66%)
LY-P2	42,255,300	36,751,239 (86.97%)	34,866,620 (82.51%)	1,884,619 (4.46%)
LY-P3	40,585,448	36,039,552 (88.80%)	34,563,939 (85.16%)	1,475,613 (3.64%)
LY-P4	41,598,832	37,237,663 (89.52%)	35,708,403 (85.84%)	1,529,260 (3.68%)
HY-P1	45,354,578	39,353,441 (86.77%)	37,548,846 (82.79%)	1,804,595 (3.98%)
HY-P2	43,711,718	38,673,900 (88.47%)	36,969,638 (84.58%)	1,704,262 (3.90%)
HY-P3	45,166,294	40,695,963 (90.10%)	39,014,043 (86.38%)	1,681,920 (3.72%)
HY-P4	40,658,890	36,305,854 (89.29%)	34,837,942 (85.68%)	1,467,912 (3.61%)

Q20, sequencing error rates lower than 1%; Q30, sequencing error rates lower than 0.1%; GC, the percentage of G and C bases in all transcripts; N, unknown base. *LY-H* Hypothalamic samples of low-yielding group, *HY-H* Hypothalamic samples of high-yielding group, *LY-P* Pituitary samples of low-yielding group, *HY-P* Pituitary samples of high-yielding group

### Principal component analysis (PCA) and correlation analysis

**Fig. 1:**
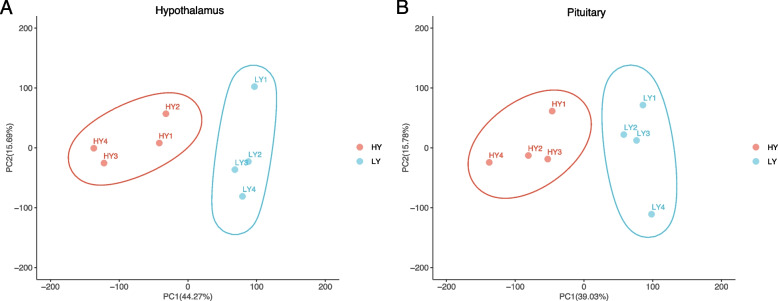
PCA score plot of (**A**) Hypothalamus transcriptomes; **B** Pituitary transcriptomes. LY, low-yielding group; HY, high-yielding group

**Fig. 2:**
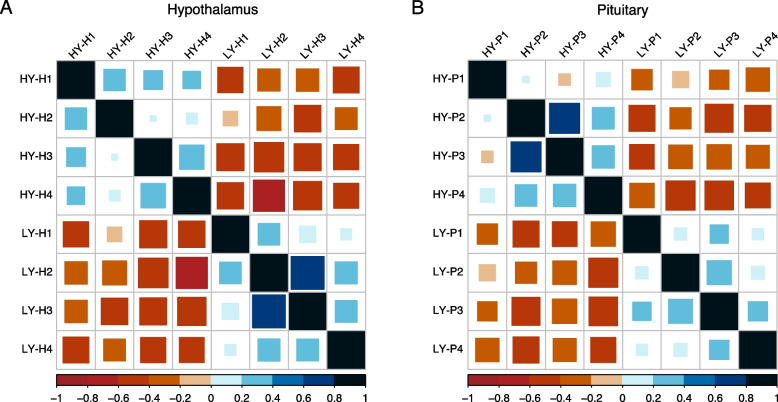
Samples correlation analysis. **A**, Hypothalamus; **B**, Pituitary. LY, low-yielding group; HY, high-yielding group

The HY and LY samples were divided into two parts in the PCA score plots for both the hypothalamic and pituitary transcriptomes (Fig. 1). PC1 and PC2 explained 59.69% of the total variation in the data of hypothalamic transcriptomes and 54.81% of the total variation in the data of pituitary transcriptomes. Consistent with the PCA results, good intra-group correlations were observed in both the hypothalamic and pituitary transcriptomes (Fig. 2).

### Differentially expressed genes

**Fig. 3:**
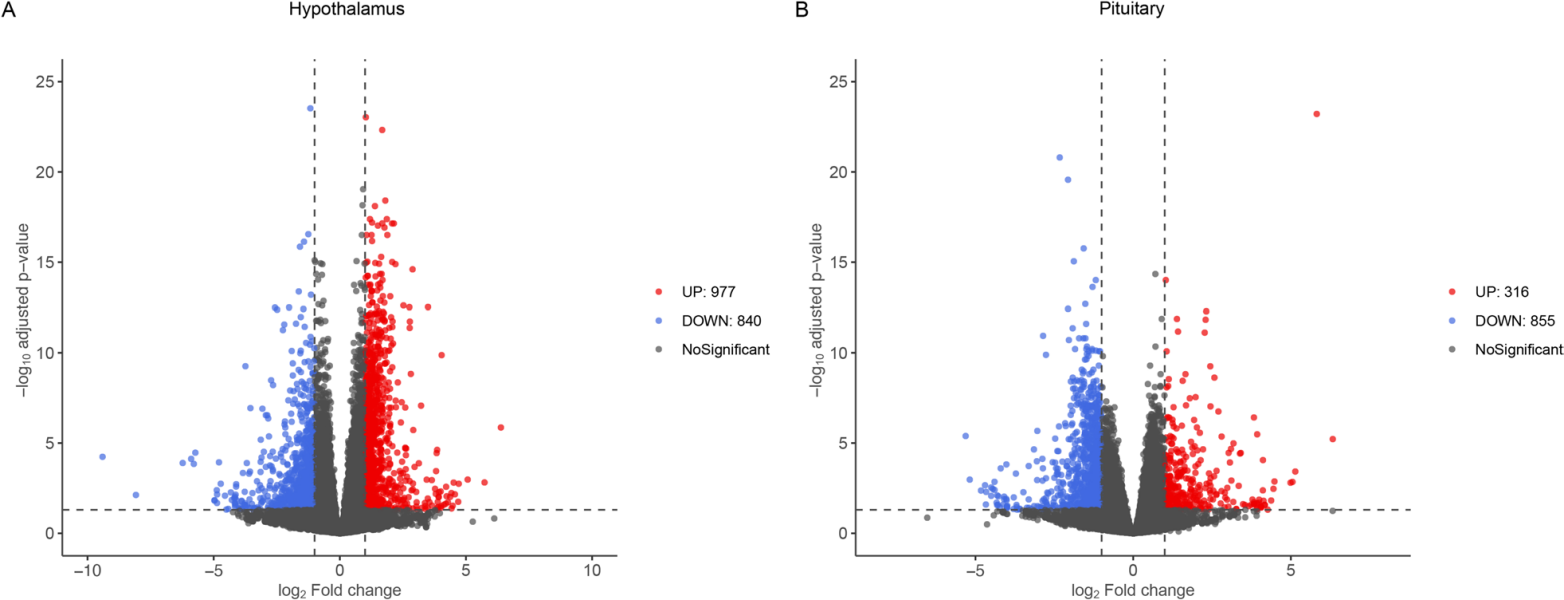
Volcano plot. **A**, Hypothalamus; **B**, Pituitary. The red plots represent significantly upregulated genes; the blue plots represent significantly downregulated genes; the gray plot represents genes with no significance

**Fig. 4:**
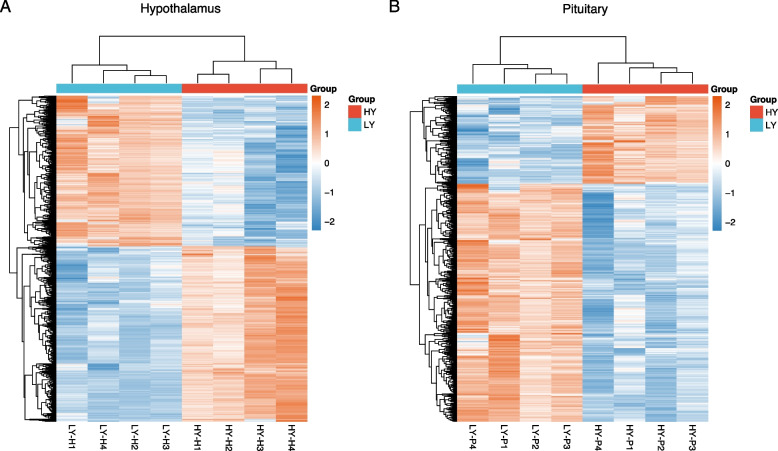
Hierarchical clustering analysis of DEGs. **A**, Hypothalamus; **B**, Pituitary. LY, low-yielding group; HY, high-yielding group

For the hypothalamus, a total of 1,817 DEGs were identified, including 977 upregulated genes and 840 downregulated genes in the HY samples (Fig. 3A, Table S1). For the pituitary, a total of 1,171 DEGs were identified, including 316 upregulated genes and 855 downregulated genes in the HY samples (Fig. 3B, Table S2). Hierarchical clustering analysis of the DEGs showed that samples from the same group clustered together, and the heatmap visually reflected the differences in gene expression patterns between the different groups (Fig. 4).

**Fig. 5:**
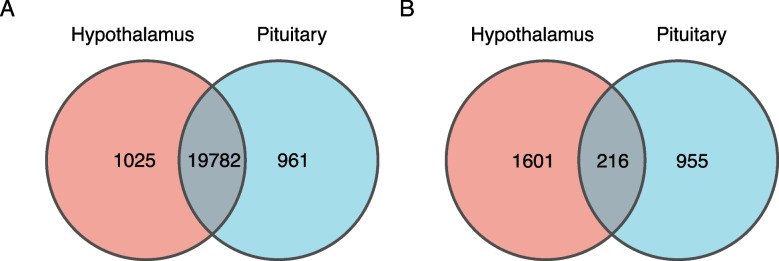
Venn diagrams. **A**, All genes identified; **B**, Differentially expressed genes

Venn diagrams showed that 19,782 genes were expressed in both the hypothalamus and pituitary (Fig. 5A). A total of 216 DEGs were identified in both tissues (Fig. 5B), including 19 DEGs upregulated in both tissues of the HY group (Table 4). These DEGs were mainly associated with ion transport and channel (*KCNG4*, *KCNC4*, and *KCNV1*), signal transduction (*PARD6A*, *CREB3L3*, *OTP*, *MIOX*, *CNTN6*, *GPR68*, *SSTR4*, *CARTPT*, *NMS*, *GAD2*, *CPLX1*, and *TAFA1*), and neuronal migration (*NCAN* and *TUBA1A*). In addition, 18 DEGs were downregulated in both tissues of the HY group, which were mainly associated with ion transport and channel, signal transduction, and immunity.

**Table 4:** DEGs upregulated in both hypothalamus and pituitary of high-yielding individuals

Category	Upregulated	Downregulated
Ion transport and channel	KCNG4	SLC22A3
	KCNC4	TMC1
	KCNV1	ATP6V0A4
Signal transduction	PARD6A	PTH2R
	CREB3L3	RHO
	OTP	ARHGAP28
	MIOX	WFIKKN2
	CNTN6	SESN1
	GPR68	LEPR
	SSTR4	ADIPOQ
	CARTPT	
	NMS	
	GAD2	
	CPLX1	
	TAFA1	
Neuronal migration	NCAN	GCM2
	TUBA1A	
Immunity		F13A1
		C7
		SOCS3
		LAG3
		BG8
Methyltransferase		LRTOMT
Other	SPCS2	ELOVL3
	AGT	

### Gene set enrichment analysis

**Fig. 6:**
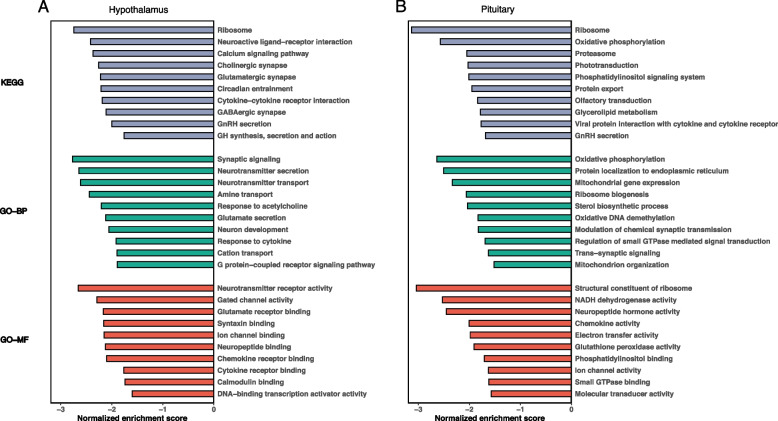
Enrichment plot. **A**, Hypothalamus; **B**, Pituitary

In the hypothalamus, a total of 71 KEGG pathways were significantly enriched (Table S3). The top 10 enriched KEGG pathways were ribosome, neuroactive ligand-receptor interaction, calcium signaling pathway, cholinergic synapse, glutamatergic synapse, circadian entrainment, cytokine-cytokine receptor interaction, GABAergic synapse, GnRH secretion, and GH synthesis, secretion, and action (Fig. 6A). A total of 255 GO-BP terms were significantly enriched. The top 10 enriched GO-BP terms were synaptic signaling, neurotransmitter secretion, neurotransmitter transport, amine transport, response to acetylcholine, glutamate secretion, neuron development, response to cytokines, cation transport, and the G protein-coupled receptor signaling pathway. A total of 39 GO-MF terms were significantly enriched. The top 10 enriched GO-MF terms were neurotransmitter receptor activity, gated channel activity, glutamate receptor binding, syntaxin binding, ion channel binding, neuropeptide binding, chemokine receptor binding, cytokine receptor binding, calmodulin binding, and DNA-binding transcription activator activity.

In the pituitary, a total of 16 KEGG pathways were significantly enriched (Table S4). Two of the top 10 enriched KEGG pathways were the same as those in the hypothalamus, namely, ribosome and GnRH secretions, while the other eight were oxidative phosphorylation, proteasome, phototransduction, phosphatidylinositol signaling system, protein export, olfactory transduction, glycerolipid metabolism, and viral protein interaction with cytokine and cytokine receptor (Fig. 6B). A total of 49 GO-BP terms were significantly enriched. The top 10 enriched GO-BP terms were oxidative phosphorylation, protein localization to the endoplasmic reticulum, mitochondrial gene expression, ribosome biogenesis, sterol biosynthetic process, oxidative DNA demethylation, modulation of chemical synaptic transmission, regulation of small GTPase-mediated signal transduction, trans-synaptic signaling, and mitochondrion organization. A total of 32 GO-MF terms were significantly enriched. One of the top 10 enriched GO-MF terms, i.e., ion channel activity, was also identified in the hypothalamus. The other nine of the top 10 enriched GO-MF terms were structural constituent of ribosome, NADH dehydrogenase activity, neuropeptide hormone activity, chemokine activity, electron transfer activity, glutathione peroxidase activity, phosphatidylinositol binding, small GTPase binding, and molecular transducer activity.

### qRT-PCR validation of RNA-Seq results

**Fig. 7:**
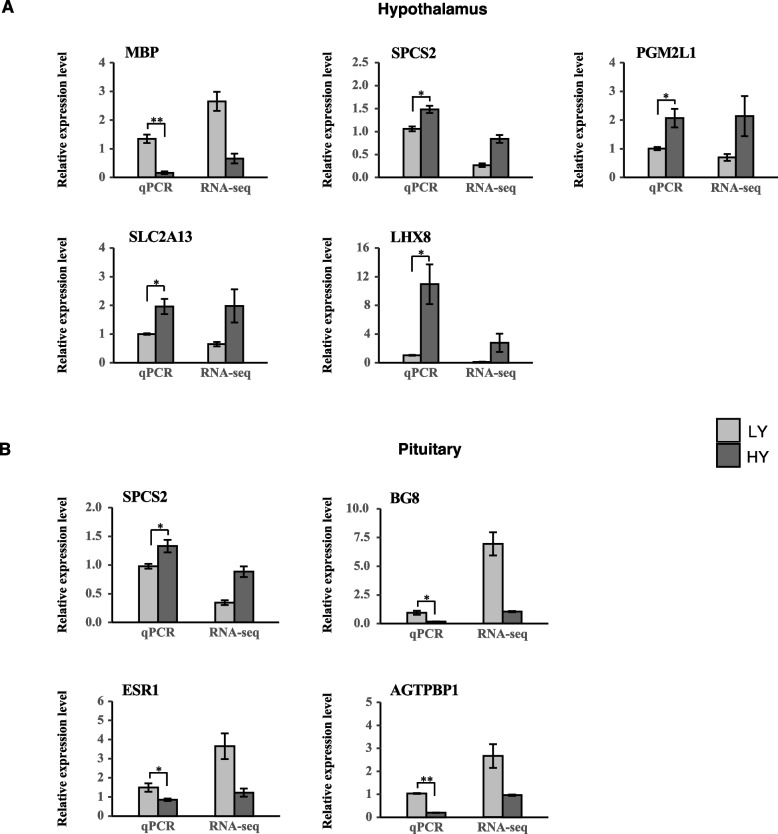
qRT-PCR validation of differentially expressed genes identified in transcriptome sequencing. The relative expression levels of genes were calculated according to the 2^−ΔΔCt^ method using β-actin as an internal reference RNA. *, *P* < 0.05; **, *P* < 0.01. LY, low-yielding group; HY, high-yielding group

To validate the RNA-seq results, five DEGs (*MBP*, *SPCS2*, *PGM2L1*, *SLC2A13*, and *LHX8*) in the hypothalamus and four DEGs (*SPCS2*, *BG8*, *ESR1*, and *AGTPBP1*) in the pituitary were selected for qRT-PCR analysis. The results showed that the expression trends determined via qRT-PCR were consistent with the RNA-Seq results (Fig. 7), indicating that the RNA-seq results were reliable.

## Discussion

Egg production traits are determined by ovarian function and regulated by the HPO axis. We previously investigated the ovarian transcriptome of Changshun green-shell chicken [1]; therefore, in the present study, we investigated the hypothalamic and pituitary transcriptomes of the same individuals. The transcriptome quality assessment and alignment indicated that our transcriptome data were reliable and suitable for subsequent analyses. Additionally, the PCA and correlation analysis indicated obvious differences in gene expression in the hypothalamus and pituitary between HY and LY individuals. Furthermore, the results of qRT-PCR indicated that the analyses of transcriptome data were reliable.

GnRH is the central neuroendocrine regulator of the HPO axis and has been shown to affect egg production performance in chicken [7, 8]. In our previous study, the GnRH signaling pathway was enriched in the ovarian tissue [1]. As expected, the KEGG pathway GnRH secretion was enriched in both the hypothalamus and pituitary in the present study. Similar results were reported by Wu et al. [9], who compared the pituitary and hypothalamic microRNA transcriptomes between low- and high-yielding Luhua chickens. With regard to DEGs, three ion transport and channel-related genes, *KCNG4*, *KCNC4*, and *KCNV1*, were upregulated in both the hypothalamus and pituitary of HY individuals. *KCNG4*, *KCNC4*, and *KCNV1* all belong to the voltage-gated potassium channel gene family, which has diverse functions, such as regulating neurotransmitter release, insulin secretion, neuronal excitability, epithelial electrolyte transport, and cell volume. In avian, the effects of voltage-gated potassium channels on the HPO axis are not clear. However, in mice, Pielecka-Fortuna et al. [10] found that voltage-gated potassium channels participate in the estrogenic regulation of GnRH. A similar result was reported by DeFazio and Moenter [11]. Our study thus suggested that *KCNG4*, *KCNC4*, and *KCNV1* might also play similar roles in chickens.

The HPO axis is regulated by specific neuropeptide-expressing neurons that sense signals from afferent neurons, resulting in the activation of a wide variety of signal transduction cascades [12]. Signal transduction between two neurons relies mainly on neurotransmitters released by synapses, including acetylcholine, glutamate, and GABA. GABA has been shown to be involved in avian egg production. Chen et al. [13] found that the transcript level of GABA-A receptor (*GABRA*) was markedly upregulated in chicken ovarian follicles from low-yielding hens compared to that in high-yielding hens. Luan et al. [14] reported that the expression level of *GABRA* mRNA was downregulated in ovaries during the laying period when compared with the non-laying period in geese. The roles of acetylcholine and glutamate in avian egg production have not been reported, but they have been shown to participate in the regulation of the HPO axis in mammals [15–17]. In the present study, the KEGG pathways cholinergic synapse, glutamatergic synapse, and GABAergic synapse were enriched in the hypothalamus, suggesting the important roles of these neurotransmitters in the regulation of the HPO axis in chickens. Calcium is a vital element for neurotransmitter release [18], and in the present study, the KEGG pathway calcium signaling pathway was enriched in the hypothalamus. In addition to neurotransmitter release, another KEGG pathway, the neuroactive ligand-receptor interaction pathway, which comprises multiple receptors that are associated with cell signaling, was enriched in the hypothalamus [19, 20]. Li et al. [21] compared 175 high-quality RNA-seq samples of the hypothalamus, pituitary, ovary, and testis from four poultry (chicken, duck, pigeon, and goose) and four mammals (human, cattle, pig, and sheep), and suggested that the neuroactive ligand-receptor interaction pathway was critical in reproductive divergence between poultry and mammals. In fish, Wang et al. [22] found that the neuroactive ligand-receptor interaction pathway could affect steroid hormone synthesis in the gonads through the HPO axis. Similar result was reported by Tian et al. [23]. In our previous study, we identified the ovarian steroidogenesis pathway as one of the most enriched pathways in the ovaries of high yielding Changshun green-shell laying hens [1]. Taken together, the present study suggests that the neuroactive ligand-receptor interaction pathway may affect ovarian sex steroid hormone synthesis through the HPO axis in chickens. In the pituitary, two signal transduction related KEGG pathways, phototransduction and olfactory transduction, were enriched. Lighting is well known to play important roles in avian egg production, whereas the involvement of smell in egg production is not clear [24]. In fruit flies, Dweck et al. [25] found that the olfactory sensory pathway is necessary for egg-laying behavior. Thus, the olfactory transduction pathway may paly a similar role in chickens. In accordance with the KEGG enrichment results, several neurotransmitter and synaptic signaling-related GO terms were also enriched in the hypothalamus or pituitary.

The present study also identified several signal transduction-related genes that are upregulated in both the hypothalamus and pituitary of HY individuals. *Gad2* encodes GAD65, which is preferred in presynaptic terminals for the synthesis of GABA for vesicle release [26]. In accordance with the results of the present study, Mishra et al. [2] reported that *Gad2* was upregulated in the pituitary of high yielding Chinese Luhua laying hens. Neuromedin S (*NMS*) and cocaine- and amphetamine-regulated transcripts (*CARTPT*) are two neuropeptides that are widely distributed in the central nervous system (CNS). Although their roles in avian egg production are not clear, they have been shown to participate in signal transduction pathways in estrogenic feedback regulation on the HPO axis in mammals [27, 28]. Our study thus suggests that *NMS* and *CARTPT* might play similar roles in chicken. G protein-coupled receptors (GPCRs) are a large family of cell surface receptors that share a common structure and signaling method. Some GPCR members, such as GPR54, GPR101, and GPR173, have been shown to work as the receptor of GnRH and other reproductive neurons [12]. In the present study, *GPR68*, a proton-sensing GPCR that responds to extracellular acidity and regulates a variety of cellular functions, was upregulated in both tissues of HY individuals. Meanwhile, *CREB3L3*, a downstream gene of reproductive neurons—GPCR pathways, is also upregulated in HY individuals [29, 30]. Thus, our study suggests that *GPR68* might act on the HPO axis through *CREB3L3* in chicken. SSTR4 is a somatostatin receptor (SSTR) that is commonly believed to have antithetical functions with SSTR2 in the CNS [31]. SSTR2 mediates the inhibition of electrical excitability of GnRH neurons in mice [32]. Thus, SSTR4 might regulate the HPO axis by promoting the electrical excitability of GnRH neurons. The other signal transduction-related genes identified in the present study have not been reported to be involved in the regulation of the HPO axis. Further studies are necessary to investigate their possible roles.

Circadian rhythms regulate diverse behavioral and physiological processes, including metabolism, food intake, sleep, immunity, and endocrine functions [33]. Many animal studies have demonstrated that circadian rhythm-related genes influence both male and female fertility by regulating the estrus cycle, LH surge, sperm production and maturation, and insemination and fertilization timing [34]. In chickens, Zhang et al. [35] showed that clock genes in the oviduct play direct roles in the infundibulum and uterus. In ducks, Tao et al. [36] reported that circadian rhythms of the ovary may help regulate ovulation. In the present study, KEGG pathway circadian entrainment was enriched in the hypothalamus, suggesting that the hypothalamic clock may be a key factor affecting egg production.

We compared our results with those of Mishra et al. [2] and Wang and Ma [5], who also sequenced the hypothalamus and pituitary transcriptomes between high- and low-yielding hens. However, no same DEGs, enriched pathways, or GO terms were found between any two of the three studies, probably due to the differences in breed, age, and sampling time. Further studies are needed to confirm the effects of these factors, especially the sampling time, on gene expression in the HPO axis.

In conclusion, we characterized and evaluated the hypothalamic and pituitary transcriptomes in LY and HY Changshun green-shell laying hens. We identified 19 DEGs that were upregulated in both the hypothalamus and pituitary gland, and these could provide an important reference for the molecular breeding of Changshun green-shell laying hens. Our results suggest that GnRH secretion, signal transduction, especially neurotransmitter release, and hypothalamic rhythms may play crucial roles in the regulation of egg production.

### Supplementary Information

**Additional file 1: Table S1. **DEGs in hypothalamus.

**Additional file 2: Table S2. **DEGs in pituitary.

**Additional file 3: Table S3. **GSEA of hypothalamic transcriptome.

**Additional file 4: Table S4. **GSEA of pituitary transcriptome.

## Data Availability

The datasets generated and analysed during the current study are available in the Genome Sequence Archive (Genomics, Proteomics & Bioinformatics 2021) in National Genomics Data Center (Nucleic Acids Res 2022), China National Center for Bioinformation / Beijing Institute of Genomics, Chinese Academy of Sciences (GSA: CRA012172) that are publicly accessible at https://ngdc.cncb.ac.cn/gsa.

## Abbreviations

FAO: Food and agriculture organization of the united nations
HPO: Hypothalamus-pituitary-ovarian
GnRH: Gonadotropin-releasing hormone
LH: Luteinizing hormone
FSH: Follicle-stimulating hormone
GnIH: Gonadotropin-inhibitory hormone
GH: Growth hormone
HY: High-yield
LY: Low-yield
DEGs: Differentially expressed genes
KEGG: Kyoto Encyclopedia of Genes and Genomes
GO: Gene Ontology
qRT-PCR: Real-time quantitative PCR
PCA: Principal component analysis
GO-BP: Gene Ontology Biological Process
GO-MF: Gene Ontology Molecular Function
GABA: γ-aminobutyric acid
NMS: Neuromedin S
CARTPT: Cocaine and amphetamine-regulated transcripts
CNS: Central nervous system
GPCRs: G protein-coupled receptors
SSTR: Somatostatin receptor
